# First case report of a percutaneous coronary intervention with intracoronary lithotripsy in a heavily calcified and tortuous right coronary artery using the R-One^+^ robotic system

**DOI:** 10.1093/ehjcr/ytae563

**Published:** 2024-10-21

**Authors:** Milad Golabkesh, Diana Mundfortz, Michael Haude

**Affiliations:** Department of Cardiology, Heart and Vascular Center, Rheinland Klinikum Neuss, Preussenstr 84, Neuss 41464, Germany; Department of Cardiology, Heart and Vascular Center, Rheinland Klinikum Neuss, Preussenstr 84, Neuss 41464, Germany; Department of Cardiology, Heart and Vascular Center, Rheinland Klinikum Neuss, Preussenstr 84, Neuss 41464, Germany

**Keywords:** Coronary artery disease, Calcified right coronary artery, Tortuous access, Robotic percutaneous coronary intervention, Intracoronary lithotripsy, Case report

## Abstract

**Background:**

Advancement in interventional techniques has significantly improved the ability of percutaneous coronary intervention (PCI) to treat complex coronary artery disease. Despite these advancements, coronary artery calcification poses a substantial challenge during PCI, contributing to increased risks of procedural complications, prolonged procedure duration, and an increase in radiation exposure dose for both patients and physicians. Recently, robotic PCI has emerged, allowing physicians to remotely control and deliver wires and catheters, leading to a notable reduction of the operator radiation exposure and a decrease in the risk of operator physical injuries such as back pain.

**Case summary:**

We report the first robotic PCI with the R-One^+^™ robotic system using intracoronary lithotripsy for lesion preparation of two heavily calcified lesions in a tortuous right coronary artery of a 60-year-old male patient followed by double drug-eluting stent implantation.

**Discussion:**

Robotic PCI with the R-One^+^™ system can not only manage wires, balloons, or stent systems but can also precisely position more bulky catheters such as intracoronary lithotripsy catheters to the target site even in the presence of a tortuous access.

**Learning points**

- Robotic percutaneous coronary intervention with the R-One^+^™ system can be performed safely and effectively also in more complex lesions such as calcified lesions with tortuous access.
- The R-One^+^™ system allows the usage of even more bulky devices such as intracoronary lithotripsy catheters.

## Introduction

Coronary artery disease (CAD) remains the first leading cause of death worldwide among adults.^1^ Over the last two decades, advancements in interventional techniques, including adjunctive methods of lesion preparation, have notably enhanced the ability of percutaneous coronary intervention (PCI) in addressing complex anatomical CAD.^2^ Despite technique advancements, coronary artery calcification remains a major challenge during PCI. It is associated with an elevated risk of periprocedural complications,^3,4^ prolonged procedural duration, increased patient and physician’s radiation exposure, and physical burden.^5^ Recently, robotic PCI (R-PCI) demonstrated safety and efficacy even in more complex lesions allowing physicians to remotely control and precisely deliver wires, balloon catheters, and stent delivery systems.^6^ This approach leads to a significant reduction in radiation exposure as well as a protection against physical injuries such as back pain for the operator.^7^ To date, we report the first R-PCI with the one and only currently available and CE-marked R-PCI system, the R-One^+^™ system, using intracoronary lithotripsy (IVL) for plaque modification in the treatment of a heavily calcified and tortuous right coronary artery (RCA) lesion.

## Summary figure

Overview of the R-PCI. R-One^+^™ system setting: (*A*) telemanipulated robotic unit mounted with a single-use sterile cassette that includes a catheter path, a guidewire path, and a standby path. (*B*) Command unit located in the control room, outside of the catheterization laboratory. The command unit houses the catheter joystick and the guidewire joystick allowing remote control and delivering of devices loaded into the robot. IVUS, intravascular ultrasound; IVL, intracoronary lithotripsy; NC, non-compliant.

**Figure:**
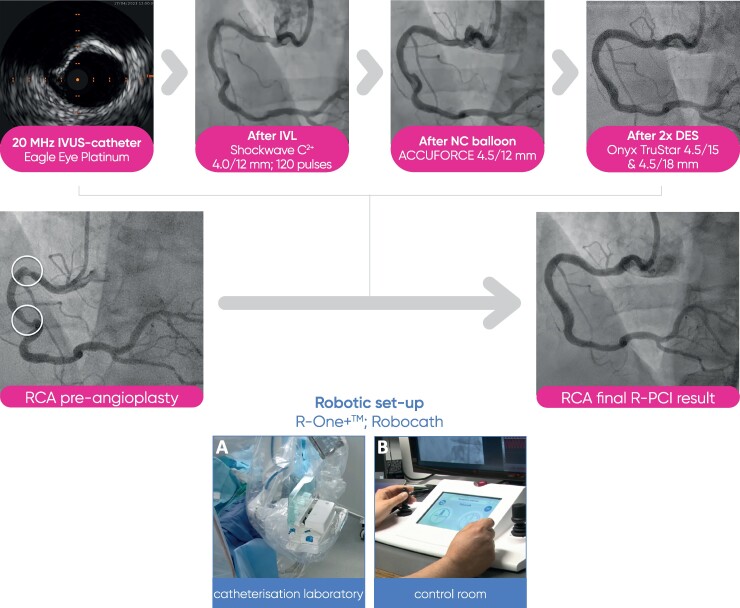


## Case summary

**Figure 1:**
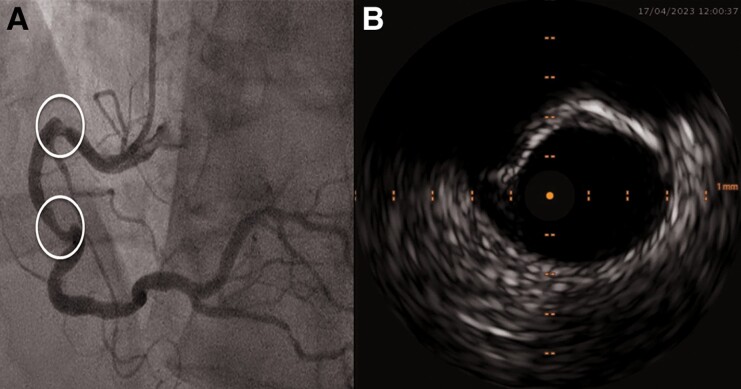
(*A*) Index right coronary artery lesion angiography. (*B*) Intravascular ultrasound of the proximal right coronary artery stenosis.

A 60-year-old male patient with a history of prior smoking (40 packs-years), hypertension, hyperlipidaemia, and a family predisposition for CAD was referred to our centre with acute coronary syndrome (ACS) reporting acute angina pectoris in the absence of previous angina symptoms. Prior medication included atorvastatin and amlodipine. Upon admission to our department, no pathological findings were observed on the initial physical examination. The electrocardiogram showed a sinus rhythm with horizontal ST-segment depression in leads II, III, and aVF. The echocardiographic findings indicated a normal left ventricular ejection fraction (LVEF) of 55% with inferolateral hypokinaesia, a good right ventricular function, and no evidence of relevant valve pathologies or pericardial effusion. The 0/1-h algorithm using high-sensitive troponin T (TnT-hs, Roche Elecsys^®^) met the criteria (0-h TnT-hs: 306 pg/mL; 1-h TnT-hs: 754 pg/mL) for non-ST elevation myocardial infarction (NSTEMI). Coronary angiography was performed on the same day and revealed a two-vessel CAD with diffuse sclerosis, subtotal stenosis of the intermediate branch (identified as the culprit lesion for the NSTEMI). Additionally, there were two highly calcified and tortuous American College of Cardiology (ACC)/American Heart Association (AHA) type B lesions^8,9^ located at segments 1 and 2 of the RCA (*Figure 1A*). For further RCA evaluation, intravascular ultrasound (IVUS) evaluation was performed using the 20 MHz Philips IVUS catheter (EagleEye Platinum). However, the operator could not pass the IVUS catheter across the first RCA stenosis because of tortuous access and suspected severe calcification (*Figure 1B*). Intravascular ultrasound evaluation of the accessible segment showed a minimal lumen area of 2.8 mm^2^ with a proximal reference vessel diameter of 5 mm and an eccentric stenosis showing a 90° arc of calcium.

Consecutive intermediate branch PCI was performed non-robotically using a right radial approach. After 6-French guiding catheter insertion and wiring of the intermediate branch (ASAHI Sion^®^ blue), lesion preparation included non-compliant (NC, ACCUFORCE 2.5 × 12 mm) and scoring balloon (AngioSculpt 3.0 × 10 mm) dilatation, followed by IVUS-evaluated drug-eluting stent (DES) implantation (Onyx TruStar 2.75 × 22 mm) and post-dilatation (ACCUFORCE 3.0 × 12 mm) with good angiographic result.

We staged the treatment of the RCA lesions for Day 3 of the hospital admission using a R-PCI system (R-One^+^™, Robocath, France) (Summary figure), attempting the inclusion of IVL (Shockwave Medical, Inc., Santa Clara, CA, USA) to allow calcified plaque modification followed by stent implantation.

**Figure 2:**
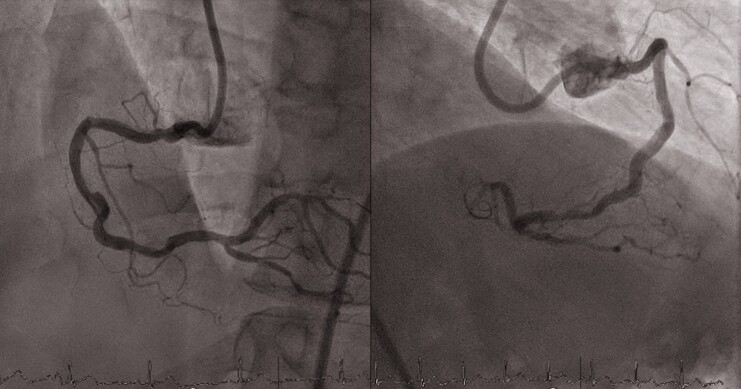
Right coronary artery intracoronary lithotripsy results.

**Figure 3:**
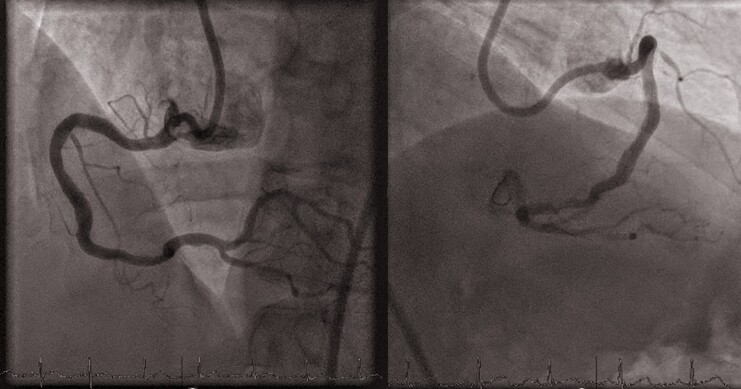
Robotic percutaneous intervention final results.

The procedure started with 8-French guiding catheter insertion to the RCA ostium via right femoral approach. Then, the R-One^+^™ system was connected to the Y-connector and then fixed to the cassette. After robotic wiring of the RCA (ASAHI Sion^®^ blue), an IVL balloon (4/12 mm, Shockwave C^2+^, Shockwave Medical) was placed robotically using the ‘R-Precise mode’ (millimetre advancement mode) through the tortuous proximal access and proximal stenosis to the distal lesion. Lesion preparation was performed with serial inflations of the coronary IVL balloon from the distal to the proximal part of the lesion at 4 atm with application of 10 pulses at each application, followed by inflation of the IVL balloon to 6 atm. A total of 12 cycles with 10 pulses were applied (4 cycles in the distal lesion and 8 cycles in the proximal lesion). Final results after IVL are shown in *Figure 2*. Thereafter, additional pre-dilatation was performed with a NC balloon (4.5/12 mm, ACCUFORCE at 12 atm) inflated from distally to proximally, positioned by the R-One^+^™ system. Then two DES were positioned and implanted first distally and then proximally (4.5/18 and 4.5/15 mm DES, Onyx TruStar™, Medtronic, 16 atm, respectively) using the robot’s ‘R-Precise mode’. For post-dilatation, a NC balloon (5.0/8 mm, ACCUFORCE at 18 atm) was robotically positioned and then inflated. The final angiography showed good implant results (*Figure 3*). Advancement, retraction, and positioning of all devices were successfully achieved by the R-One^+^™ system without any manual assistance. No complication occurred. The duration of the procedure was 62 min, fluoroscopy time 16 min. A total of 240 mL of contrast agent was used, of which 120 mL was applied to achieve stable guiding catheter engagement due to tortuous vessel access. Furthermore, patient radiation exposure was 6357.11 Gycm^2^ and the primary operator radiation exposure was 0 µSv as all the procedure was performed from the control room located outside of the catheterization laboratory. Postinterventional outcomes were uneventful. The patient was discharged on Day 4 after the RCA intervention. Outpatient follow-up after 4 weeks revealed no episodes of angina.

## Discussion

We report the first case of a successful PCI with IVL in a heavily calcified and tortuous RCA using the one and only currently available and CE-marked robotic system for coronary application, the R-One^+^™ system. The intervention was effective and safe without any complication.

The presence of coronary calcific plaques and coronary tortuosity have a negative impact on PCI outcome parameters such as stent expansion, stent apposition, and edge dissection leading to a higher rate of target vessel failure.^8,9^ The use of IVL offers a safe and effective method of lesion preparation to facilitate and optimize stent implantation in severely calcified lesions.^10^ The simultaneous use of R-PCI and IVL combines the robotic-guided accuracy of wire manipulations and precise catheter placement with the mechanism of action of IVL. Particularly, our case demonstrated that the robotic advancement and removal of the 4/12 mm IVL balloon (Shockwave C2+, Shockwave Medical) as a more bulky device (crossing profile range from 0.044 to 0.047 in) using the ‘R-Precise mode’ worked without any difficulties. Of note, despite tortuous access, robotic wire and balloon catheter management was feasible without any guiding catheter dislodgement.

Due to the generally more favourable profile of the IVL catheter and better trackability due to the absence of a bulky transducer, the IVL catheter represents an overall more favourable crossability compared to the Philips 20 MHz IVUS EagleEye Platinum catheter (crossing profile of 0.0459 in), which could not cross the first RCA stenosis.

Of note, the current R-One^+^™ system does not allow a robotic manipulation of the guiding catheter. In case of catheter dislodgement, the interventional operator has to manually reposition the guiding catheter. In conclusion, R-PCI with the R-One^+^™ system might offer an effective option for the treatment of complex lesions with severe calcification in tortuous vessels by also using bulkier catheter systems.

## Data Availability

The data underlying this article will be shared on reasonable request to the corresponding author.

## Lead author biography

Since 2006, Professor Michael Haude is the head of Medical Clinic I at Lukaskrankenhaus Neuss and, since 2006, the director of the Heart and Vascular Center at the Rheinland Klinikum Neuss, a teaching hospital of the Heinrich-Heine University Düsseldorf. His research interests focus on the interventional therapy of coronary artery disease, including intravascular ultrasound, bioresorbable scaffolds, complex percutaneous coronary interventions, structural heart disease intervention, and complication management strategies. He is the former President of the European Association of Percutaneous Cardiovascular Interventions (EAPCI) from 2016 to 2018 and former Board Member of the European Society of Cardiology (ESC).

## Notes

**Consent:** In accordance with CARE guidelines, patient informed consent was obtained for the publication of the case.

**Funding:** None.
